# Effect of High-Pressure Micro-Fluidization on the Inactivation of *Staphylococcus aureus* in Liquid Food

**DOI:** 10.3390/foods12234306

**Published:** 2023-11-29

**Authors:** Zequn Zhang, Tianlin Cui, Luyang Tai, Kangyi Mu, Yicong Shi, Fang Chen, Xiaojun Liao, Xiaosong Hu, Li Dong

**Affiliations:** 1 National Engineering Research Center for Fruit and Vegetable Processing, Key Laboratory of Fruits and Vegetables Processing, College of Food Science and Nutritional Engineering, Ministry of Agriculture, Beijing 100083, China; zqzhang1995@126.com (Z.Z.); tianlincui@163.com (T.C.); 18013885232@126.com (L.T.); mkys1999@163.com (K.M.); s20223061139@cau.edu.cn (Y.S.); chenfangch@sina.com (F.C.); liaoxjun@cau.edu.cn (X.L.); huxiaos@263.net (X.H.); 2 Engineering Research Centre for Fruits and Vegetables Processing, Ministry of Education, China Agricultural University, No. 17, East Qinghua Road, Haidian District, Beijing 100083, China

**Keywords:** high-pressure micro-fluidization, *Staphylococcus aureus*, inactivation effect, antibacterial mechanism, apple juice

## Abstract

High-pressure homogenization has been extensively studied for its excellent homogenization effect and the prospect of continuous liquid food production, but its sterilization ability still needs to be improved. In this study, we replaced the homogenization valve with two opposing diamond nozzles (0.05 mm inner diameter) so that the fluid collided at high velocity, corresponding to high-pressure micro-fluidization (HPM). Moreover, HPM treatment significantly inactivated *Staphylococcus aureus* ~7 log in the liquid with no detectable sub-lethal state at a pressure of 400 MPa and a discharge temperature of 50 °C. The sterilization effect of HPM on *S. aureus* subsp. *aureus* was attributed to a significantly disrupted cell structure and increased membrane permeability, which led to the leakage of intracellular proteins, resulting in bacterial death. At the same time, HPM treatment was able to significantly reduce the ability of *S. aureus* subsp. *aureus* to form biofilms, which, in turn, reduced its virulence. Finally, compared to the simulated system, more effective sterilization was observed in apple juice, with its color and pH remaining unchanged, which suggested that HPM can be used to process other liquid foods.

## 1. Introduction

Foodborne illness caused by food poisoning through microorganisms plays a key role in the increase in morbidity and mortality around the world [1]. The most common foodborne illnesses are diarrheal diseases, which cause 550 million people to fall ill and even 230,000 deaths every year [2], with *Staphylococcus aureus* as one of the main pathogenic bacteria. *S. aureus* can survive in potentially dry and stressful environments and is recognized as one of the major commensal and opportunistic foodborne pathogens in food, including milk, juice, and meat [1,3]. Food poisoning and foodborne illness caused by *S. aureus* remain a threat to food safety and public health, especially its biofilm formation, which can enhance bacterial resistance and produce more toxin [4]. Therefore, its control in the food industry continues to be a worldwide problem [3].

Recently, non-thermal processing technology, including high hydrostatic pressure and high-pressure carbon dioxide, has been widely used to maintain the sensory quality and nutritional properties of foods significantly [5,6]. But they cannot achieve the continuous production of liquid food products, resulting in low efficiency and high energy consumption. Currently, high-pressure homogenization technology (HPH) for liquid foods is becoming one of the most popular non-thermal food processing technologies due to its advantages, such as continuous production with a short processing time [7], homogenization to improve bioavailability [8], and sterilization. HPH inactivated 3.42 log *E. coli* and 2.21 log *S. aureus* at 200 MPa in PBS with 40 °C inlet temperature [9], yet ~7 log *E. coli* K-12 could be killed at 300 MPa with 70 °C [10]. So, the sterilization effect of HPH on microorganisms was related to the treatment pressure, temperature, and viscosity of the liquid [11]; however, the details of bacterial inactivation mechanism by HPM have been obscure.

In addition, HPH technology at 100–200 MPa was applied to juice processing, which not only reduced the microbial content but also slowed the occurrence of browning [12,13], thereby improving the shelf life of apple juice [14]. And similar results were found in organic kiwifruit juice and carrot juice [15,16]. Thus, HPH appeared to be a favorable means of balancing quality, safety, and production efficiency. However, processing conditions in past studies ranging up to 200 MPa even for three cycles inactivated only about ~3 log of microorganism, which, in turn, required the product to be stored at 4 °C, resulting in higher costs [15].

In this study, we converted the homogenization valve (HPH) into an orifice (high-pressure micro-fluidization, HPM) to achieve a higher homogenization pressure (up to 400 MPa). And the inactivation mechanism of *S. aureus* by HPM at different pressures and temperatures was further investigated with fluorescence staining, flow cytometry, scanning electron microscopy, and other techniques. Finally, the sterilization effect of HPM on *S. aureus* in apple juice was evaluated.

## 2. Materials and Methods

### 2.1. Bacterial Strain and Growth Conditions

*S. aureus* subsp. *aureus* ATCC 6538 was stored at −80 °C in nutrient broth (NB, Beijing AoBoXing Bio-Tech Co., Ltd., Beijing, China) with 25% (*v*/*v*) glycerol, after which it was streaked on nutrient agar medium (NA, Beijing AoBoXing Bio-Tech Co., Ltd., Beijing, China) and incubated at 37 °C for 24 h. A single colony was selected and transferred into 5 mL of NB at 37 °C for 12 h at 180 rpm. The culture was then diluted in 400 mL of fresh NB at a 1:100 ratio and incubated at 37 °C for 4 h to reach mid-logarithmic growth. The culture was subsequently centrifuged at 8000× *g* and 4 °C for 10 min using a CF16RXII centrifuge (Hitachi Co., Ltd., Tokyo, Japan). The resulting pellet of *S. aureus* subsp. *aureus* was resuspended in 4 L of sterile physiological saline (PS, 0.85% NaCl).

### 2.2. High-Pressure Micro-Fluidization Treatment

**Figure 1:**
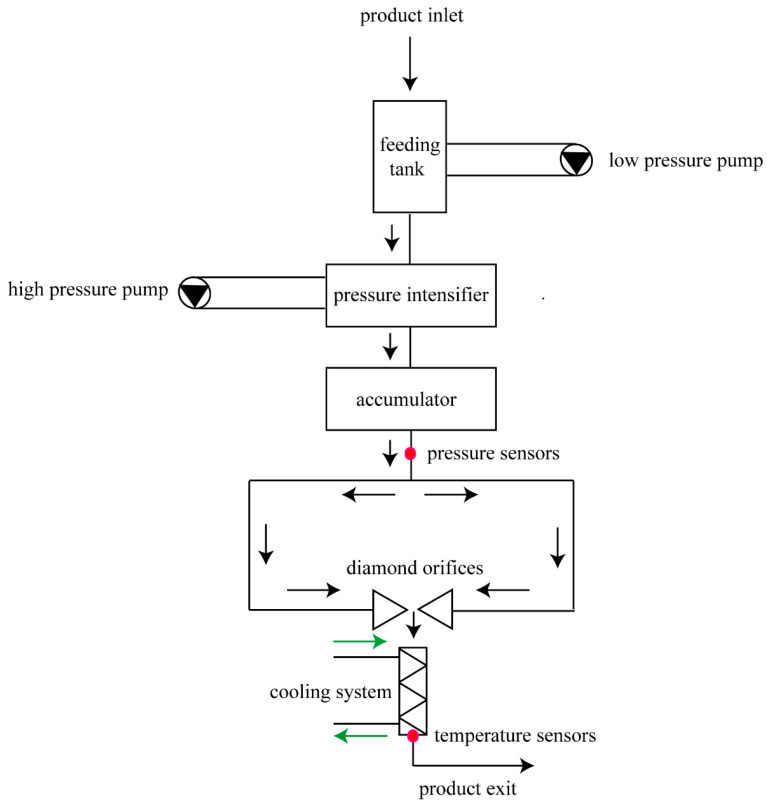
The sketch map of high-pressure micro-fluidization.

The HPM device used in the experiment was self-designed (Figure 1) and consisted of a power supply (triple-phase asynchronous motor, 11 kW, 50 Hz, Yancheng Cat motor manufacturing Co., Ltd., Yancheng, China) and pressurizing system, impingement zone, temperature control system, and data acquisition system. The impingement zone consisted of a cylinder and two parallel diamond nozzles (inner diameter 0.05 mm, distance 2 mm) wrapped in food-grade stainless steel that was embedded into a cylinder. The sample was pressurized, passed through the diamond nozzle to form an ultrahigh-velocity jet, and collided in the impingement zone. A heat exchanger was then used immediately downstream of the chamber to control the outlet temperature, and different discharge temperatures were achieved by adjusting the cooling temperature. Due to the different flow rates of the liquid caused by the different pressures, the residence time of the sample in the machine (from inlet to outlet) was different at different pressures: 200, 300, and 400 MPa corresponded to 4.88, 3.41, and 3.02 min, respectively.

For HPM treatment, about 4 L of bacterial broth (25 °C) was added to the product inlet and, to maintain a constant discharge temperature (25 °C/50 °C) at different pressures, the cooling temperature was set to 25 °C/50 °C for a 25 °C/50 °C discharge temperature (200 MPa), 23 °C/45 °C for a 25 °C/50 °C discharge temperature (300 MPa), and 20 °C/39 °C for a 25 °C/50 °C discharge temperature (400 MPa), according to the correspondence between the cooling temperature and the discharge temperature. After HPM treatment, the samples were pipetted into 50 mL sterile centrifuge tubes and immediately analyzed. Samples without HPM treatment were used as the control.

### 2.3. Enumeration of Viable Cells and Injured Cells

The total plate count method was used to enumerate *S. aureus* subsp. *aureus* after HPM treatment. Briefly, a sample of the bacterial suspension was serially diluted with PS (1:10), and then 100 μL of the diluted or undiluted bacterial suspension was incubated on NA. Meanwhile, the bacterial suspension was also plated onto a Baird–Parker agar base (BP, Qingdao Hope Bio-Technology Co., Ltd., Qingdao, China), which is a selective medium that distinguishes viable and injured cells through their ability to degrade an egg yolk tellurite emulsion, according to previous reports [1,17]. The colonies on the plates were counted after incubation at 37 °C for 24 h. The logarithm of the surviving *S. aureus* subsp. *aureus* (log_10_ (N_t_/N_0_)) was defined as the *S. aureus* subsp. *aureus* reduction for each treatment, while N_0_ and N_t_ are the *S. aureus* subsp. *aureus* counts before and after treatment, respectively.

### 2.4. Scanning Electron Microscopy Analysis

SEM was used to observe the morphology of *S. aureus* subsp. *aureus* after treatment with HPM at different pressures and discharge temperatures (200 MPa and 25 °C/50 °C, 300 MPa and 25 °C/50 °C, and 400 MPa and 25 °C/50 °C), respectively. Logarithmic-phase cells (control) and treated cells were centrifuged at 664× *g* for 15 min, washed twice in phosphate-buffered saline (PBS, pH 7.4), and resuspended in 1 mL of pre-chilled glutaraldehyde solution (2.5%, Solarbio Biotechnology Co., Ltd., Beijing, China) at 4 °C for 12 h. Then, the glutaraldehyde solution was removed and the cell pellets were washed by PBS buffer twice, cast with 1% osmic acid for 1–2 h, and further dehydrated for 15 min with different concentrations of cold ethanol solutions (30–100%). Finally, the cells were lyophilized and coated with gold palladium. The obtained samples were observed in detail by SEM (Hitachi SU8020, Hitachi Co., Ltd., Tokyo, Japan).

### 2.5. Determination of Cell Membrane Permeability

#### 2.5.1. Fluorescence Microscopy Analysis

To evaluate the membrane permeability of *S. aureus* subsp. *aureus* after HPM treatment, fluorescence microscopy analysis was used. In brief, the samples were analyzed using a LIVE/DEAD BacLight bacterial viability kit (Invitrogen, Carlsbad, CA, USA), including two types of DNA staining dyes. One dye was STYO 9 (3.34 mM), which permeates intact cell membranes, inserts into DNA strands, and emits green fluorescence. The other dye was propidium iodide (PI, 20 mM), which only permeates cell membranes with increased permeability and then binds to DNA strands to emit red fluorescence. After HPM treatment, 1.5 μL of the reagent mixtures were added to 500 μL of the cell suspension, followed by incubation in the dark at room temperature for 15 min. After staining, 20 μL of each suspension was mounted on a microscope slide, covered with a cover slip, and then immersion oil was added. Finally, the samples were observed under a fluorescence microscope (Axio Observer A1, Carl Zeiss, Germany) with excitation wavelengths of 488 and 536 nm for STYO 9 and PI, respectively. The relative fluorescence intensity was quantified by ImageJ software (version 1.48).

#### 2.5.2. Measurement of Bacterial Intracellular Protein Loss

The loss of bacterial intercellular protein with HPM treatment (200 MPa and 25 °C/50 °C; 300 MPa and 25 °C/50 °C; 400 MPa and 25 °C/50 °C) was determined by an existing method with minor modifications [18]. After HPM treatment, the sample was centrifuged (3000 rpm) for 10 min, and the precipitated *S. aureus* subsp. *aureus* was washed and resuspended in sterile distilled water. Cells were disrupted by sonication at 300 W for 10 min on the ice. The protein concentration was measured by a BCA protein assay kit (Jiancheng Bioengineering Institute, Nanjing, China) according to the user manual, and sodium dodecyl sulfate-polyacrylamide gel electrophoresis (SDS-PAGE) analysis was also employed to monitor the leakage and integrity of proteins. Log-phase bacteria were used as the control. Specifically, 20 μL of protein samples were mixed with 5 μL of 5× SDS sample buffer in a PCR tube. The mixture was then heated to 100 °C for 5 min to denature the proteins. Subsequently, 20 μL of the denatured protein mixture were added to each well of the configured 10% separating gel. Once the electrophoresis was completed, the gel was stained using Coomassie Brilliant Blue Fast Staining Solution (Solarbio Biotechnology Co., Ltd., Beijing, China).

### 2.6. Flow Cytometry Analysis

FCM was performed according to a previously reported method with minor modifications [19]. After staining as in Section 2.5.1, the dye-stained cells were immediately analyzed with flow cytometry (LSR Fortessa, BD Biosciences, Franklin Lakes, NJ, USA). SYTO 9 fluorescence was quantified with FITC channel at 530 nm and PI fluorescence was quantified with PE-A channel at 635 nm. All samples were evaluated after 30,000 events were recorded. The log-phase cells were used as the control. Flow cytometry data were analyzed using FlowJo ver. 10.8.1 software (Becton Dickinson Inc, Franklin Lakes, NJ, USA.).

### 2.7. Analysis of the Biofilm Formation Ability

Crystal violet (CV) staining, as described previously using microtiter plates [20], was performed for determining the biofilm formation ability of *S. aureus* subsp. *aureus* after HPM treatment. Briefly, 10 μL of HPM-treated cells and 190 μL of fresh TSBG buffer (tryptic soy broth with 1% glucose, Beijing AoBoXing Bio-Tech Co., Ltd., Beijing, China) were added into the wells of 96-well plates (Corning Incorporated-Life Science, Suzhou, China), which were placed in a stationary incubator at 37 °C for 12–48 h to develop biofilms. Then, the wells were carefully washed twice with PBS to remove suspended bacteria and allowed to dry overnight at 4 °C. The biofilms were stained with 200 μL of a 0.1% CV solution (Solarbio Biotechnology Co., Ltd., Beijing, China) for 5 min at 37 °C. After staining, the wells were washed twice with PBS to remove the unbound CV solution and dried at 37 °C. Finally, dissolution of cells with adhered CV was achieved with 200 μL of ethanol, and the fluorescence was measured at 595 nm with a SpectraMax iD5 microplate reader (Molecular Devices, San Jose, CA, USA).

### 2.8. Application of HPM in Apple Juice

The inactivation effect of HPM on *S. aureus* subsp. *aureus* in apple juice and the effect of HPM treatment on apple juice quality was evaluated. The sterilized apple juice was procured from a local supermarket in Beijing, China, and it is produced by Beijing Huiyuan Beverage and Food Group Co., Ltd (Beijing, China). Then, before inoculation, we determined by plate counting that apple juice was sterile. Ten times the amount of *S. aureus* subsp. *aureus* pellets harvested according to Section 2.1 were resuspended in 4 L of apple juice. After the HPM treatment, the viable cells were counted as described in Section 2.3, and the color of the apple juice was determined with a CR-400 Minolta Chroma meter (Konica Minolta, Osaka, Japan). For each sample, three measurements of L*, a*, and b* were taken. The L* value indicates brightness, while the +a* and −a* values represent redness and greenness, respectively. Similarly, the +b* and −b* values correspond to yellowness and blueness, respectively. The pH of the samples was determined using a Sartorius PB-10 pH meter (Sartorius, Gottingen, Germany). All analyses were conducted at room temperature, and each experiment was conducted in triplicate.

### 2.9. Statistical Analysis

All analyses were performed in IBM SPSS Statistics 22. The significance level (*p*) was 0.05. Data in figures were presented as the mean ± standard deviation of three replicates. Figure generation and data fitting were performed in GraphPad Prism 8. Every treatment was carried out in duplicate and each sample was analyzed in triplicate.

## 3. Results

### 3.1. Reduction Effect of HPM on S. aureus *subsp.* aureus

**Figure 2:**
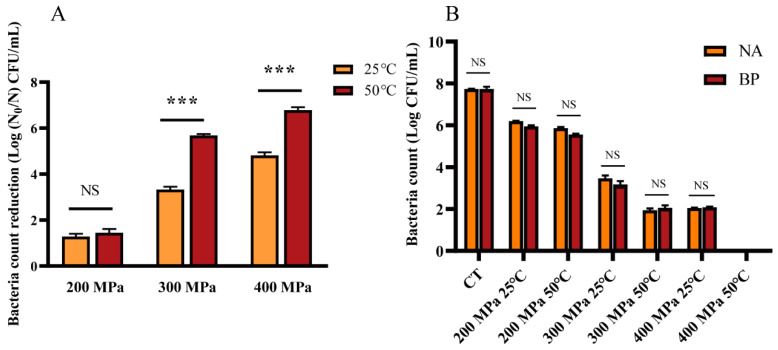
Inactivation effect of HPM on *S. aureus* subsp. *aureus*. (**A**) Growth of *S. aureus* subsp. *aureus* on NA plate after HPM treatment. (**B**) Growth of *S. aureus* subsp. *aureus* on NA and BP plates after HPM treatment. Significance was calculated using the *t*-test (NS, *p* > 0.05, *** *p* < 0.05).

To explore the effect of HPM on *S. aureus* subsp. *aureus*, survival and sublethal damage were detected by plating. The inactivation number of *S. aureus* subsp. *aureus* by HPM is shown in Figure 2A. When the discharge temperature was 25 °C, then, 1.28, 3.33, and 4.82 log cells were inactivated at 200, 300, and 400 MPa, respectively. When the discharge temperature rose to 50 °C, the inactivation effect increased significantly to 1.45 and 5.68 log at 200 and 300 MPa, respectively. Moreover, all *S. aureus* subsp. *aureus* (>7 log) were inactivated at 400 MPa and 50 °C. At the same pressure and temperature treatment, there was no difference in the growth of *S. aureus* subsp. *aureus* on the NA and BP media (Figure 2B), which indicated that HPM treatment did not cause sublethal damage to *S. aureus* subsp. *aureus*. These results suggested that, with the increase in pressure and discharge temperature, the inactivation of *S. aureus* subsp. *aureus* became more effective.

**Figure 3:**
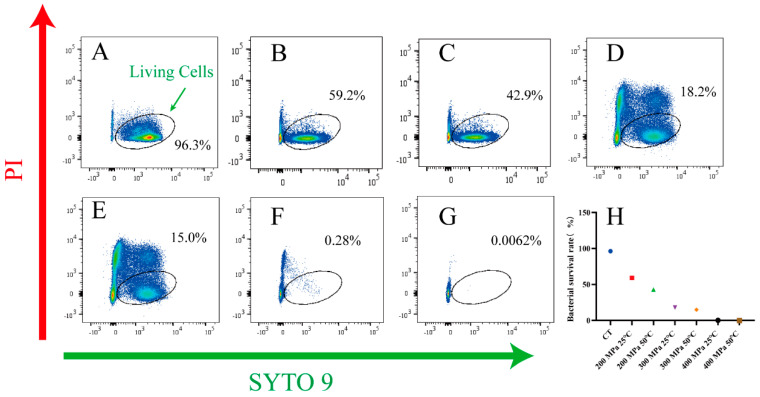
The flow cytometry images of *S. aureus* subsp. *aureus* cells subjected to HPM. (**A**) The living cells (control sample); (**B**) samples treated with HPM at 200 MPa/25 °C; (**C**) samples treated with HPM at 200 MPa/50 °C; (**D**) samples treated with HPM at 300 MPa/25 °C; (**E**) samples treated with HPM at 300 MPa/50 °C; (**F**) samples treated with HPM at 400 MPa/25 °C; (**G**) samples treated with HPM at 400 MPa/50 °C; (**H**) percentage of living cells after HPM treatment.

Moreover, FCM was used to determine the number of live *S. aureus* subsp. *aureus* cells after HPM treatment. The results are presented in Figure 3. The circle representing the live cells was determined according to log-phase cells (Figure 3A). As the treatment pressure increased, cells enriched in the circle region moved to the other regions, implying a gradual decrease in the live cells. When the discharge temperature was 25 °C, the pressure increased from 200 MPa to 400 MPa and the live cells decreased from 59.2 to 0.28% (Figure 3B,D,F). At a discharge temperature of 50 °C, the number of live cells tended to decrease with increasing pressure (Figure 3C,E,G). Even at 400 MPa, only 0.0062% of the live cells were located in the circle region (Figure 3G). These points in Figure 3H represent the percentage of live cells under different treatments, which exhibited the same trend as the FCM results, as shown in Figure 3A–G. In summary, the higher the pressure and temperature, the fewer the number of live cells in the circle, which indicated that the inactivation effect of HPM treatment on *S. aureus* subsp. *aureus* increased with increasing pressure and temperature.

### 3.2. Analysis of Cell Morphology

**Figure 4:**
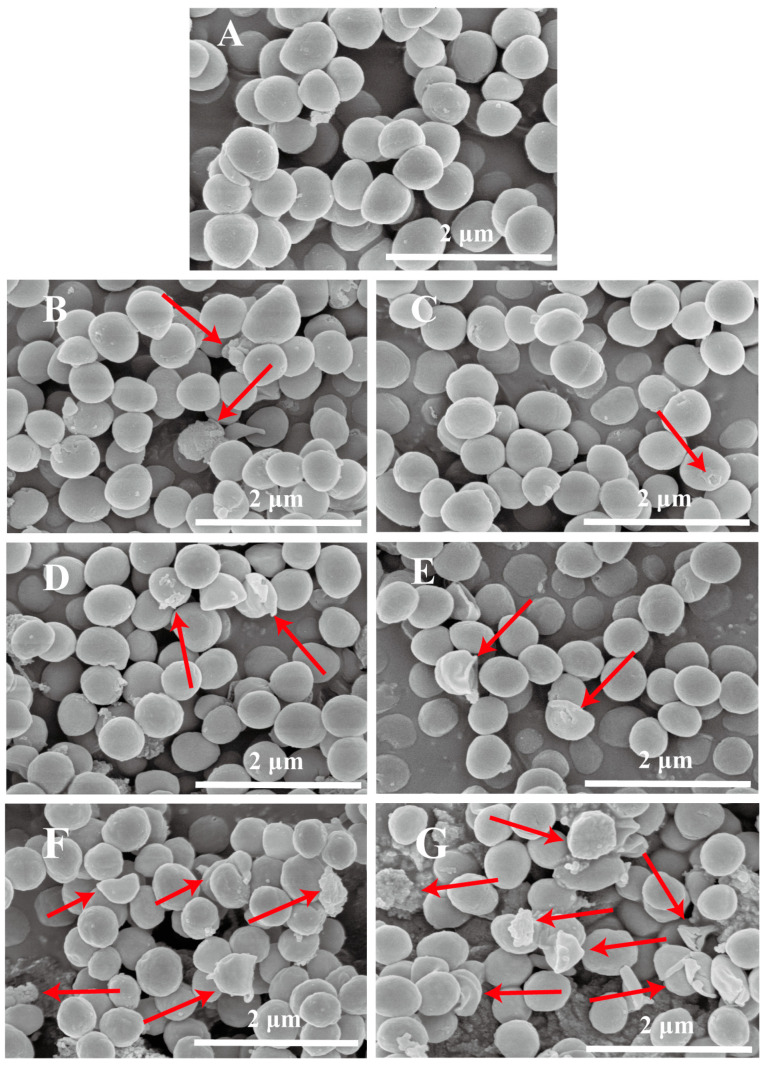
Scanning electron microscope images of *S. aureus* subsp. *aureus* after HPM treatment. (**A**) The log-phase cells (control sample); (**B**) samples treated with HPM at 200 MPa/25 °C; (**C**) samples treated with HPM at 200 MPa/50 °C; (**D**) samples treated with HPM at 300 MPa/25 °C; (**E**) samples treated with HPM at 300 MPa/50 °C; (**F**) samples treated with HPM at 400 MPa/25 °C; (**G**) samples treated with HPM at 400 MPa/50 °C. The red arrows point to the damage.

To investigate the destruction of HPM on the bacterial structure, the cell morphology of *S. aureus* subsp. *aureus* was analyzed using SEM. As shown in Figure 4A, the membrane structure of untreated *S. aureus* subsp. *aureus* was intact, the surface was smooth, and the shape was regular and spherical. After HPM treatment, the *S. aureus* subsp. *aureus* morphology changed significantly with the increased pressure. With treatment at 200 MPa (Figure 4B,C), the surface of the bacteria began to become rough with some areas showing slight breakage, and, in particular, some holes were found in the membrane. After 300 MPa treatment (Figure 4D,E), the cell surface roughness increased and the cell morphology was more severely disrupted; for example, some large breaks in the membrane were observed. Eventually, at 400 MPa (Figure 4F,G), cells were significantly ruptured and a large number of fragments were observed. All these results indicated that the number of damaged cells increased significantly, and the cell morphology was destroyed, especially the membrane structure, with increasing pressure and temperature.

### 3.3. Analysis of Cell Membrane Integrity

**Figure 5:**
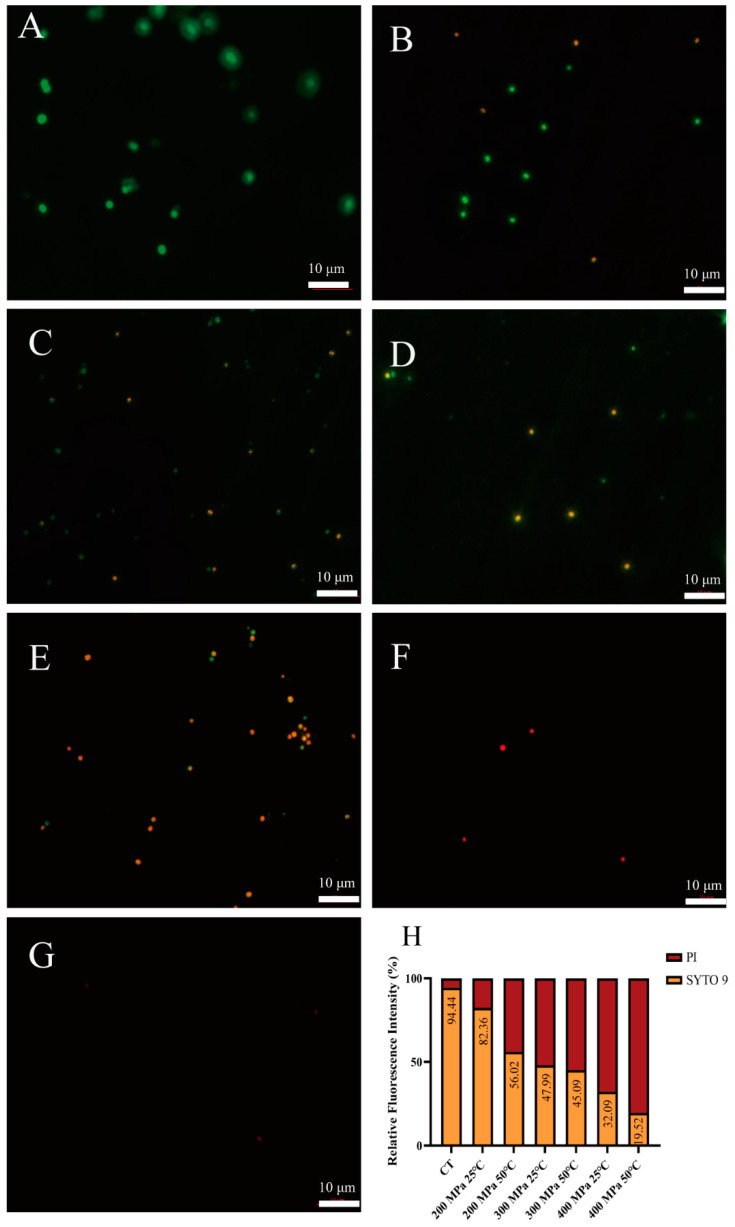
Fluorescence microscopy images of *S. aureus* subsp. *aureus* treated by HPM. (**A**) The live cells (control sample); (**B**) samples treated with 200 MPa/25 °C; (**C**) samples treated with 200 MPa/50 °C; (**D**) samples treated with 300 MPa/25 °C; (**E**) samples treated with 300 MPa/50 °C; (**F**) samples treated with 400 MPa/25 °C; (**G**) samples treated with 400 MPa/50 °C; (**H**) relative fluorescence intensity of fluorescence microscopy of HPM—treated *S. aureus* subsp. *aureus*.

Furthermore, fluorescence microscopy was used to evaluate the changes in the membrane structure of HPM—treated *S. aureus* subsp. *aureus*. Stained cells with intact and damaged membranes showed green and red fluorescence, respectively, as the cell membrane permeability increased. As shown in Figure 5A, the untreated cells with an intact membrane showed green fluorescence. After treatment at 200 MPa and 25 °C, only a limited number of bacteria were red (Figure 5B). When the pressure increased, the bacteria gradually stained red (yellow, 300 MPa, Figure 5D; red, 400 MPa, Figure 5F). Similar results were shown at 50 °C. Interestingly, it was difficult to find intact cells in the field of view after 400 MPa treatment (Figure 5G). Figure 5H shows that, with increasing pressure and temperature, the fluorescence intensity of PI (red) gradually increased and that of SYTO 9 (green) gradually decreased. The fluorescence microscopy results confirmed that the changes in membrane permeability of *S. aureus* subsp. *aureus* cells were pressure- and temperature—dependent.

### 3.4. Analysis of Intracellular Protein

**Figure 6:**
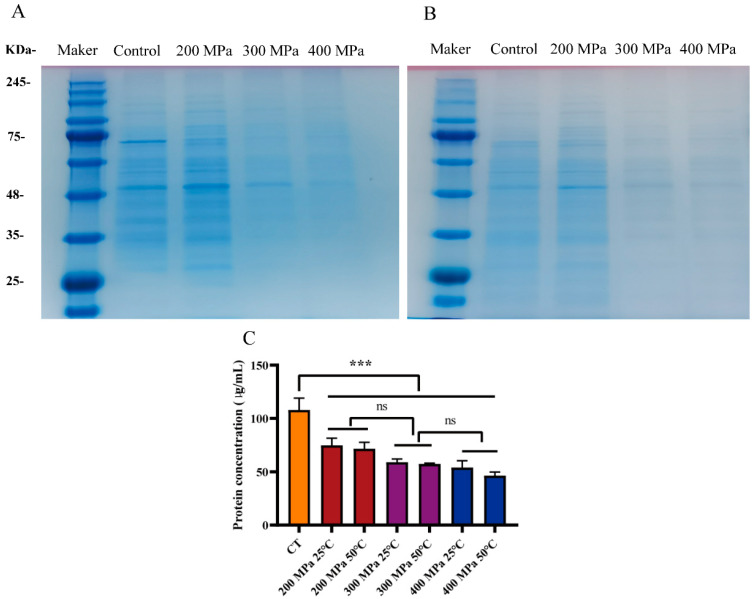
Determination of cell intracellular proteins by SDS-PAGE in *S. aureus* subsp. *aureus* with HPM treatment. (**A**) Protein bands of *S. aureus* subsp. *aureus* at different pressure and 25 °C; (**B**) protein bands of *S. aureus* subsp. *aureus* at different pressure and 50 °C; (**C**) intracellular protein concentrations in *S. aureus* subsp. *aureus* with different treatments. Significance was calculated using the *t*-test (ns, *p* > 0.05, *** *p* < 0.05).

Protein is essential for the survival of bacteria. It not only serves as the backbone of the cell membrane but also participates in various biochemical reactions in the cell [18,21]. The protein fractions in bacterial cells were measured using SDS-PAGE. As shown in Figure 6, the protein concentration of *S. aureus* subsp. *aureus* treated with HPM decreased significantly (lightening of electrophoretic bands), especially when the pressure was 300 and 400 MPa. The protein concentration was quantified (Figure 6) and the intracellular protein of *S. aureus* subsp. *aureus* treated with HPM was 46–75 μg/mL, which was much lower than that of the control group (107.98 μg/mL). Therefore, the HPM treatment impacted the intracellular protein of *S. aureus* subsp. *aureus*, which was attributed to the damaged cell membrane and subsequent protein leakage.

### 3.5. Analysis of Resistance

**Figure 7:**
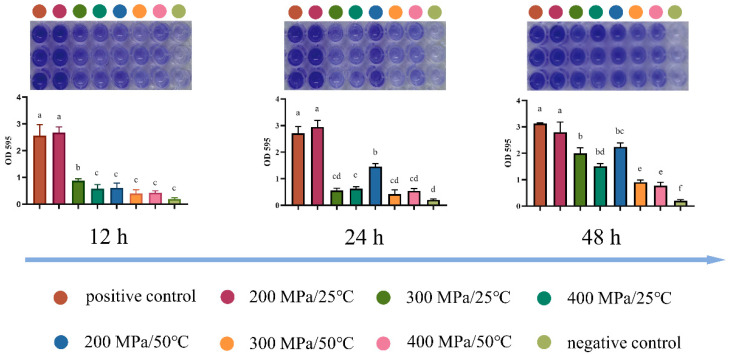
Biofilm formation capacity of *S. aureus* subsp. *aureus* with different treatments. The different letters in the same figure indicate significant differences (*p* < 0.05).

The biofilms of *S. aureus* subsp. *aureus* protect cells not only from any antimicrobial treatment but also from the immune response of the host and represent a serious threat to the food industry and human health [22]. To evaluate the potential threat of *S. aureus* subsp. *aureus* after HPM treatment, the amount of biofilm formed after different times was measured by the crystal violet staining method. As shown in Figure 7, the treated cells exhibited a significant reduction in biofilm formation compared with the control (*p* < 0.05), and the biofilm formation capacity decreased with increasing temperature and pressure (from 200 to 400 MPa) conditions. With an increasing incubation time, biofilm formation was observed in all treatment groups but was still significantly less than the positive control (*p* < 0.05) at 400 MPa and 50 °C, even after 48 h incubation. This implied that HPM treatment significantly reduced the biofilm formation capacity, which can decrease the toxicity of *S. aureus* subsp. *aureus*.

### 3.6. The Antibacterial Effect of HPM in Apple Juice

To explore the effect of HPM treatment on liquid food, apple juice was used as a simulation system to investigate the antibacterial activity and color change with HPM treatment. The results in Table 1 show that, compared with the inoculated group (8.46 log), the number of bacteria in the experimental groups after HPM treatment at 25 °C decreased to 5.36 log (200 MPa), 2.99 log (300 MPa), and 1.98 log (400 MPa). When the discharge temperature was 50 °C, *S. aureus* subsp. *aureus* was only detected at 200 MPa (2.09 log). The results showed that HPM exhibited a significant antibacterial effect on apple juice contaminated with *S. aureus* subsp. *aureus*.

Then, the color and pH of the apple juice treated by HPM were analyzed. According to Table 1, there was a significant difference in color between the fresh and inoculated groups (*p* < 0.05), which was affected by the high concentration of bacteria. The L* values of HPM—treated apple juice showed fluctuations but no significant changes after HPM, regardless of the pressure and temperature applied (*p* > 0.05), as compared with the inoculated group. As for a* and b*, their values were between those of the fresh and inoculated groups, which implied that the apple juice inoculated with the bacteria approached the color of the fresh group after HPM treatment. Furthermore, the pH of apple juice was relatively stable between 3.87 and 3.89 before and after HPM treatment, and there was no significant difference (*p* > 0.05). These results suggest that HPM treatment in acidic juices is a more effective treatment than in a simulation system.

## 4. Discussion

In recent years, HPM has been widely studied as an emerging non-thermal processing technology due to its continuous operation and enhanced preservation of nutritional properties. In fruit and vegetable processing, HPM is primarily used for homogenization and enzyme inactivation [23]. However, HPM was not found to be very effective for sterilization in the past, inactivating only 1–4 log of *S. aureus* with single-pass treatment [24,25,26]. In this study, for the first time and by using a diamond nozzle with an inner diameter of 0.05 mm as a homogenizing orifice valve, we inactivated ~7 log of *S. aureus* with single-pass treatment at a moderate temperature of 50 °C. The inactivation mechanism of *S. aureus* was also investigated.

HPM was shown to have a better inactivation effect in this study than those in previous studies. Here, a pressure of 400 MPa and a discharge temperature of 50 °C resulted in the complete killing of about 7 log of *S. aureus*. Even at 300 MPa and 50 °C, about 6 log of *S. aureus* were inactivated. In a previous report, 2 log of *S. aureus* were inactivated at a pressure of about 250 MPa and 0 °C [27]. We believe that, in addition to the slightly lower temperature and pressure, the effect of collision in our equipment contributed substantially to the inactivation of bacteria. Moreover, we found that the effect of discharge temperature on the inactivation of *S. aureus* was not significant at 200 MPa (*p* > 0.05), while, at high pressure, there was a significant difference in the inactivation effect between different discharge temperatures (*p* < 0.001). We attributed this to (1) higher pressures damaging cells more and making them more sensitive to temperature and (2) higher pressures generating more heat, which also contributed to sterilization.

Moreover, HPM-induced inactivation of *S. aureus* was observed to be lethal. There was no difference in the growth of *S. aureus* in either selective or non-selective media (*p* > 0.05), which meant there was no sublethal state. These results were consistent with previous studies [28,29,30]. However, even under the most intense conditions (400 MPa and 50 °C), intact cells were still visible with SEM, which seemed to contradict the plating results. Therefore, we suspected that, under treatment conditions of 400 MPa and 50 °C, although some of the cells were morphologically intact, their membrane permeability increased and leakage of their contents led to their death. A similar inactivation mechanism was also reported previously, in which *S. aureus* was treated with an induced electric field for 14 s and was unable to grow on plates, even though some cells were morphologically intact [31]. Furthermore, the results of fluorescence microscopy and SDS-PAGE also confirmed this finding. The red fluorescence indicating an increase in membrane permeability became stronger with increasing pressure but, at 400 MPa and 50 °C, the fluorescence was difficult to detect. This meant that almost no nucleic acid was left in the cell because both SYTO 9 and PI are fluorescent dyes that bind to nucleic acids. Furthermore, the change in intracellular protein content is also strong evidence for the membrane-destructive effect of HPM. The intracellular protein content significantly decreased with the increased pressure (*p* < 0.05), but there was no difference in intracellular protein content between different temperatures at the same pressure (*p* > 0.05). This indicated that the various physical effects caused by pressure were the main cause of intracellular protein leakage, and temperature had a limited effect on this. Lin et al. [18] also demonstrated that cell membrane permeability increased in *E. coli* after PEF treatment by using SDS-PAGE. Therefore, the membrane structure of the cell was destroyed by HPM treatment. HPM treatment caused conditions of high turbulence and shear stress, combined with compression, increased temperature, acceleration, a pressure drop, and impact forces (the relative velocity almost doubled), resulting in leakage of intracellular soluble proteins, which is an important cause of cell death. This effect is above previous reports; Donsì et al. showed that, after 10 passes, *E. coli* was inactivated by 2 log at 100 MPa, 3.5 log at 200 MPa, and 4 log at 300 MPa [32]. Furthermore, HPH treatment not only disrupted the structure of *E. coli* but also increased its susceptibility to antimicrobial peptides. This synergistic effect occurred immediately upon HPH treatment [33].

However, the results of the ability to form biofilms made us consider further. We found that HPM treatment significantly reduced the ability of *S. aureus* to form biofilms (*p* < 0.05), even under incomplete inactivation conditions, implying that the resistance of HPM-treated *S. aureus* was greatly reduced [20]. However, after 48 h of incubation at 400 MPa and 50 °C, a small amount of biofilm was still detected, which was not consistent with the plating results. FCM results explained this phenomenon. At 400 MPa and 50 °C, 0.0062% of the cells were still alive but they were unable to grow on the plate, implying that they may be transitioning to a viable but non-culturable (VBNC) state. Under optimal temperature and nutrient conditions, this fraction of cells recovered and formed biofilms, but the number of cells and their activity was very low and, therefore, toxicity was limited. This result was similar to a previous report [34], in which the bacteria, treated by starvation at low temperatures, lost the ability to grow on solid agar and required resuscitation in liquid media.

Finally, we applied the HPM technique to apple juice, and *S. aureus* subsp. *aureus* was already undetectable at a processing pressure of 300 MPa and a discharge temperature of 50 °C, far below the simulated system (400 MPa). Another study also showed that *S. aureus* was more likely to be inactivated after HPH treatment in apple juice than in PBS, with 2.33 and 2.21 log of inactivation at 200 MPa, respectively [9]. We suspect that the greater inactivation of *S. aureus* subsp. *aureus* in apple juice than in the simulated system was due to the lower pH of apple juice. Moreover, these results were much better than those already reported, in which only a 1.4 log of bacteria reduction at 200 MPa/35 °C in apple juice was achieved [14]. In another study, only a 2.5 log reduction in bacteria was achieved at 300 MPa and 20 °C in apple juice [35]. In contrast, we inactivated 8 log *S. aureus* subsp. *aureus* at 300 MPa and 50 °C.

The evaluation regarding the color and pH of the apple juice showed that HPM treatment did not change the quality of the juice. The color difference between the inoculated group and the fresh group could be caused by bacteria. With HPM treatment, the color and the values of L*, a*, and b* in the treated groups were all close to those of the fresh group because HPM treatment eliminated the effects of the bacteria by disrupting the cellular structure of the bacteria. Moreover, there was no significant difference in the pH of the apple juice before and after inoculation or HPM treatment (*p* > 0.05). The above results showed that HPM maintained good color and a stable pH of the apple juice with complete inactivation of 8 log *S. aureus* subsp. *aureus* and that similar results may be found in orange or lemon juice with a similar pH. Therefore, HPM is very promising for the production of acidic juices such as apple juice, and the simulation system results also support the production of neutral liquid foods such as milk. In summary, the research and application of HPM is an important contribution to the industrial development of liquid food processing, such as fruit and vegetable juice.

## 5. Conclusions

In this study, HPM treatment showed an excellent inactivation effect on *S. aureus* subsp. *aureus* (7 log CFU/mL) at 400 MPa and 50 °C in both simulated (PS buffer) and real systems (apple juice). Moreover, the disruption of the cell structure and subsequent outflow of intracellular proteins likely contributed to the inactivation of *S. aureus* subsp. *aureus*. Finally, HPM treatment not only inactivated *S. aureus* subsp. *aureus* 8 log but also showed no significant changes in the pH or color of the apple juice. These results suggested that HPM effectively inactivated *S. aureus* subsp. *aureus* in liquid food and preserved the quality of the food. Notably, after HPM treatment (400 MPa and 50 °C), even though no sub-lethal state was detected, the possible presence of the bacterial VBNC state should be further investigated.

## Figures and Tables

**Table 1:** Bacterial counts, color, and pH of *S. aureus* subsp. *aureus* in apple juice with different treatment.

Treatments	Bacteria Count (Log CFU/mL)	Color			pH
		L*	a*	b*	
Fresh	/	27.28 ± 0.10 a	−0.87 ± 0.07 c	4.44 ± 0.05 c	3.87 ± 0.01 a
Inoculated	8.46 ± 0.02	26.15 ± 0.13 b	−1.28 ± 0.07 a	5.67 ± 0.03 a	3.89 ± 0.01 a
200 MPa/25 °C	5.36 ± 0.09	26.35 ± 0.34 b	−0.88 ± 0.03 bc	5.20 ± 0.05 b	3.89 ± 0.01 a
300 MPa/25 °C	2.99 ± 0.03	26.27 ± 0.37 b	−1.04 ± 0.06 b	5.09 ± 0.10 b	3.88 ± 0.01 a
400 MPa/25 °C	1.98 ± 0.07	25.89 ± 0.05 b	−0.94 ± 0.01 bc	5.15 ± 0.01 b	3.87 ± 0.01 a
200 MPa/50 °C	2.09 ± 0.16	25.87 ± 0.07 b	−0.97 ± 0.05 bc	5.17 ± 0.10 b	3.87 ± 0.01 a
300 MPa/50 °C	/	26.05 ± 0.50 b	−1.03 ± 0.01 b	5.27 ± 0.22 b	3.87 ± 0.01 a
400 MPa/50 °C	/	26.17 ± 0.05 b	−1.04 ± 0.02 b	5.32 ± 0.10 bc	3.87 ± 0.01 a

Note: the different letters in the same column indicate significant differences (*p* < 0.05).

## Data Availability

Data are contained within the article.

## References

[1] Shao L., Liu Y., Tian X., Yu Q., Wang H., Li X., Dai R. (2021). Inactivation and recovery of *Staphylococcus aureus* in milk, apple juice and broth treated with ohmic heating. LWT.

[2] World Health Organization (2015). Assessing Microbiological Risks in Food. https://www.who.int/activities/assessing-microbiological-risks-in-food.

[3] Shi C., Che M., Zhang X., Liu Z., Meng R., Bu X., Ye H., Guo N. (2018). Antibacterial activity and mode of action of totarol against *Staphylococcus aureus* in carrot juice. J. Food Sci. Technol..

[4] Lee J.-S., Bae Y.-M., Lee S.-Y., Lee S.-Y. (2015). Biofilm Formation of *Staphylococcus aureus* on Various Surfaces and Their Resistance to Chlorine Sanitizer. J. Food Sci..

[5] San Martín M.F., Barbosa-Cánovas G.V., Swanson B.G. (2002). Food processing by high hydrostatic pressure. Crit. Rev. Food Sci. Nutr..

[6] Garcia-Gonzalez L., Geeraerd A.H., Spilimbergo S., Elst K., Van Ginneken L., Debevere J., Van Impe J.F., Devlieghere F. (2007). High pressure carbon dioxide inactivation of microorganisms in foods: The past, the present and the future. Int. J. Food Microbiol..

[7] Martínez-Monteagudo S.I., Yan B., Balasubramaniam V.M. (2016). Engineering Process Characterization of High-Pressure Homogenization—From Laboratory to Industrial Scale. Food Eng. Rev..

[8] Donsì F., Wang Y., Li J., Huang Q. (2010). Preparation of Curcumin Sub-micrometer Dispersions by High-Pressure Homogenization. J. Agric. Food Chem..

[9] Dong P., Zhou B., Zou H., Wang Y., Liao X., Hu X., Zhang Y. (2021). High pressure homogenization inactivation of *Escherichia coli* and *Staphylococcus aureus* in phosphate buffered saline, milk and apple juice. Lett. Appl. Microbiol..

[10] Taylor T.M., Roach A., Black D.G., Davidson P.M., Harte F. (2007). Inactivation of *Escherichia coli K-12* exposed to pressures in excess of 300 MPa in a high-pressure homogenizer. J. Food Prot..

[11] Diels A.M.J., Callewaert L., Wuytack E.Y., Masschalck B., Michiels C.W. (2005). Inactivation of *Escherichia coli* by high-pressure homogenisation is influenced by fluid viscosity but not by water activity and product composition. Int. J. Food Microbiol..

[12] Donsì F., Esposito L., Lenza E., Senatore B., Ferrari G. (2009). Production of Shelf-Stable Annurca Apple Juice with Pulp by High Pressure Homogenization. Int. J. Food Eng..

[13] Saldo J., Suárez-Jacobo Á., Gervilla R., Guamis B., Roig-Sagués A.X. (2009). Use of ultra-high-pressure homogenization to preserve apple juice without heat damage. High Press. Res..

[14] Szczepańska J., Skąpska S., Marszałek K. (2021). Continuous High-pressure Cooling-Assisted Homogenization Process for Stabilization of Apple Juice. Food Bioprocess Technol..

[15] Patrignani F., Mannozzi C., Tappi S., Tylewicz U., Pasini F., Castellone V., Riciputi Y., Rocculi P., Romani S., Caboni M.F. (2019). (Ultra) High Pressure Homogenization Potential on the Shelf-Life and Functionality of Kiwifruit Juice. Front. Microbiol..

[16] Szczepanska J., Skapska S., Polaska M., Marszalek K. (2022). High pressure homogenization with a cooling circulating system: The effect on physiochemical and rheological properties, enzymes, and carotenoid profile of carrot juice. Food Chem..

[17] Baird-Parker A.C. (1962). An Improved Diagnostic and Selective Medium for Isolating Coagulase Positive Staphylococci. J. Appl. Bacteriol..

[18] Lin L., Wang X., He R., Cui H. (2019). Action mechanism of pulsed magnetic field against *E. coli* O157:H7 and its application in vegetable juice. Food Control.

[19] Zhao L., Qin X., Wang Y., Ling J., Shi W., Pang S., Liao X. (2017). CO_2_-assisted high pressure processing on inactivation of *Escherichia coli* and *Staphylococcus aureus*. J. CO_2_ Util..

[20] Yang H., Wang M., Yu J., Wei H. (2015). Aspartate inhibits Staphylococcus aureus biofilm formation. FEMS Microbiol. Lett..

[21] Zhang Y., Liu X., Wang Y., Jiang P., Quek S. (2016). Antibacterial activity and mechanism of cinnamon essential oil against *Escherichia coli* and *Staphylococcus aureus*. Food Control.

[22] Espina L., Pagan R., Lopez D., Garcia-Gonzalo D. (2015). Individual Constituents from Essential Oils Inhibit Biofilm Mass Production by Multi-Drug Resistant Staphylococcus aureus. Molecules.

[23] Singh S.V., Singh R., Verma K., Kamble M.G., Tarafdar A., Chinchkar A.V., Pandey A.K., Sharma M., Kumar Gupta V., Sridhar K. (2022). Effect of microfluidization on quality characteristics of sapodilla (*Manilkara achras* L.) juice. Food Res. Int..

[24] Briñez W.J., Roig-Sagués A.X., Herrero M.M.H., López B.G. (2007). Inactivation of Staphylococcus spp. strains in whole milk and orange juice using ultra high pressure homogenisation at inlet temperatures of 6 and 20 °C. Food Control.

[25] Diels A.M., Wuytack E.Y., Michiels C.W. (2003). Modelling inactivation of Staphylococcus aureus and Yersinia enterocolitica by high-pressure homogenisation at different temperatures. Int. J. Food Microbiol..

[26] Wuytack E.Y., Diels A.M.J., Michiels C.W. (2002). Bacterial inactivation by high-pressure homogenisation and high hydrostatic pressure. Int. J. Food Microbiol..

[27] Zhang L., Zhu C., Chen X., Xu X., Wang H. (2021). Resistance of detached-cells of biofilm formed by *Staphylococcus aureus* to ultra high pressure homogenization. Food Res. Int..

[28] Briñez W.J., Roig-Sagués A.X., Hernández-Herrero M.M., Guamis-López B. (2006). Inactivation of two strains of *Escherichia coli* inoculated into whole and skim milk by ultrahigh-pressure homogenisation. Le Lait.

[29] Roig-Sagues A.X., Velazquez R.M., Montealegre-Agramont P., Lopez-Pedemonte T.J., Brinez-Zambrano W.J., Guamis-Lopez B., Hernandez-Herrero M.M. (2009). Fat content increases the lethality of ultra-high-pressure homogenization on *Listeria monocytogenes* in milk. J. Dairy Sci..

[30] Velázquez-Estrada R.M., Hernández-Herrero M.M., López-Pedemonte T.J., Briñez-Zambrano W.J., Guamis-López B., Roig-Sagués A.X. (2011). Inactivation of *Listeria monocytogenes* and *Salmonella enterica* serovar Senftenberg 775W inoculated into fruit juice by means of ultra high pressure homogenisation. Food Control.

[31] Zhang L., Yang N., Jin Y., Xu X. (2023). Putative inactivation mechanism and germicidal efficacy of induced electric field against *Staphylococcus aureus*. Food Microbiol..

[32] Donsì F., Annunziata M., Ferrari G. (2013). Microbial inactivation by high pressure homogenization: Effect of the disruption valve geometry. J. Food Eng..

[33] Diels A.M.J., De Taeye J., Michiels C.W. (2005). Sensitisation of *Escherichia coli* to antibacterial peptides and enzymes by high-pressure homogenisation. Int. J. Food Microbiol..

[34] Auty J.M., Jenkins C.H., Hincks J., Straatman-Iwanowska A.A., Allcock N., Turapov O., Galyov E.E., Harding S.V., Mukamolova G.V. (2022). Generation of Distinct Differentially Culturable Forms of *Burkholderia* following Starvation at Low Temperature. Microbiol. Spectr..

[35] McKay A.M., Linton M., Stirling J., Mackle A., Patterson M.F. (2011). A comparative study of changes in the microbiota of apple juice treated by high hydrostatic pressure (HHP) or high pressure homogenisation (HPH). Food Microbiol..

